# Retrospective epidemiological surveillance of bovine tuberculosis in a slaughterhouse in Northeastern Brazil

**DOI:** 10.1007/s11250-026-05038-1

**Published:** 2026-04-22

**Authors:** Breno Bezerra Aragão, Vitória Coutinho de Carvalho, Luiz Guilherme Generoso Soares de Lima, Roger Henrique Sousa da Costa, José Erisvaldo Maia Júnior

**Affiliations:** 1 Universidade Federal do Cariri (UFCA), Rua Ícaro de Sousa Moreira, 126, Muriti, Crato, Ceará, CEP 63133-610 Brazil; 2 Agência de Defesa Agropecuária do Estado do Ceará - ADAGRI, Avenida Padre Cícero, KM 03, Batateira, Crato, Ceará, CEP 63133-610 Brazil

**Keywords:** Epidemiology, Meat inspection, One Health, Zoonosis

## Abstract

Bovine tuberculosis (BTB), mainly caused by Mycobacterium bovis, remains an endemic zoonosis with significant economic and public health impact, particularly in major beef-producing countries such as Brazil. This study aimed to evaluate the occurrence, epidemiological profile, and macroscopic features of BTB-like lesions identified during routine post-mortem inspection at an officially inspected slaughterhouse in Northeastern Brazil over a six-year period (2020–2025). A retrospective observational study was conducted based on official inspection records from 79,318 slaughtered cattle. Thirty-six carcasses were totally condemned due to lesions suggestive of BTB, corresponding to an overall prevalence of 0.045% (95% CI: 0.032–0.063%), with no significant temporal trend throughout the study period. Affected animals were predominantly females (61.1%), who presented significantly higher odds of condemnation compared to males (OR = 5.76; 95% CI: 2.92–11.37; *p* < 0.001), and animals older than 36 months (75%). Macroscopically, lesions were mainly granulomatous, with caseous necrosis and calcification, primarily involving the lungs, mediastinal lymph nodes, and liver, indicating chronic and frequently systemic infection. Despite the low apparent prevalence, the consistent annual detection of advanced lesions suggests sustained endemic circulation of agents compatible with bovine tuberculosis within source herds. These findings reinforce the epidemiological value of slaughterhouse surveillance as a complementary, cost-effective tool for BTB monitoring, contributing to risk assessment, guiding herd-level control strategies, and strengthening integrated actions within a One Health framework.

## Introduction

Public health is intrinsically linked to the safety of foods of animal origin, especially in countries with high levels of cattle production and slaughter, such as Brazil, which is currently the world’s largest beef exporter, supplying more than 150 countries (Brasil 2024). In this context, officially inspected slaughterhouses serve as important instruments of epidemiological surveillance, since ante- and post-mortem sanitary inspections enable the detection of diseases of zoonotic relevance through the identification and condemnation of carcasses and viscera presenting alterations compatible with diseases of sanitary concern, such as bovine tuberculosis (García-Díez et al. 2023). Bovine tuberculosis, caused by Mycobacterium bovis, is a zoonosis of historical and current relevance, associated with risks to human health and significant economic impacts resulting from partial or total condemnation of carcasses and organs during post-mortem inspection (Gebremichael 2025).

Despite the implementation of official control programs, such as the Brazilian National Program for the Control and Eradication of Brucellosis and Tuberculosis, the disease is still identified in slaughter establishments, particularly in regions with gaps in sanitary management and epidemiological surveillance (Brasil 2025). Post-mortem inspection remains the routine method for detecting lesions suggestive of tuberculosis, which are primarily identified through visual examination, palpation, and incision, especially in lungs, lymph nodes, and other viscera. These findings suggest the circulation of infectious agents compatible with bovine tuberculosis within herds and represent an alert for zoonotic transmission risks, in addition to the economic losses associated with the disposal of products deemed unfit for human consumption (Gonçalves et al. 2022). Thus, the systematic analysis of sanitary inspection data obtained from slaughterhouses receiving animals from different regions is essential to better understand the epidemiological dynamics of the disease and to support improvements in surveillance and control strategies (Pereira et al. 2020).

The aim of this study was to evaluate the occurrence of bovine tuberculosis–like lesions in carcasses and viscera inspected at a slaughterhouse in Northeastern Brazil over a six-year period, analyzing their macroscopic characteristics, distribution patterns, and epidemiological implications within a One Health context.

## Methodology

### Study period and area

**Fig. 1:**
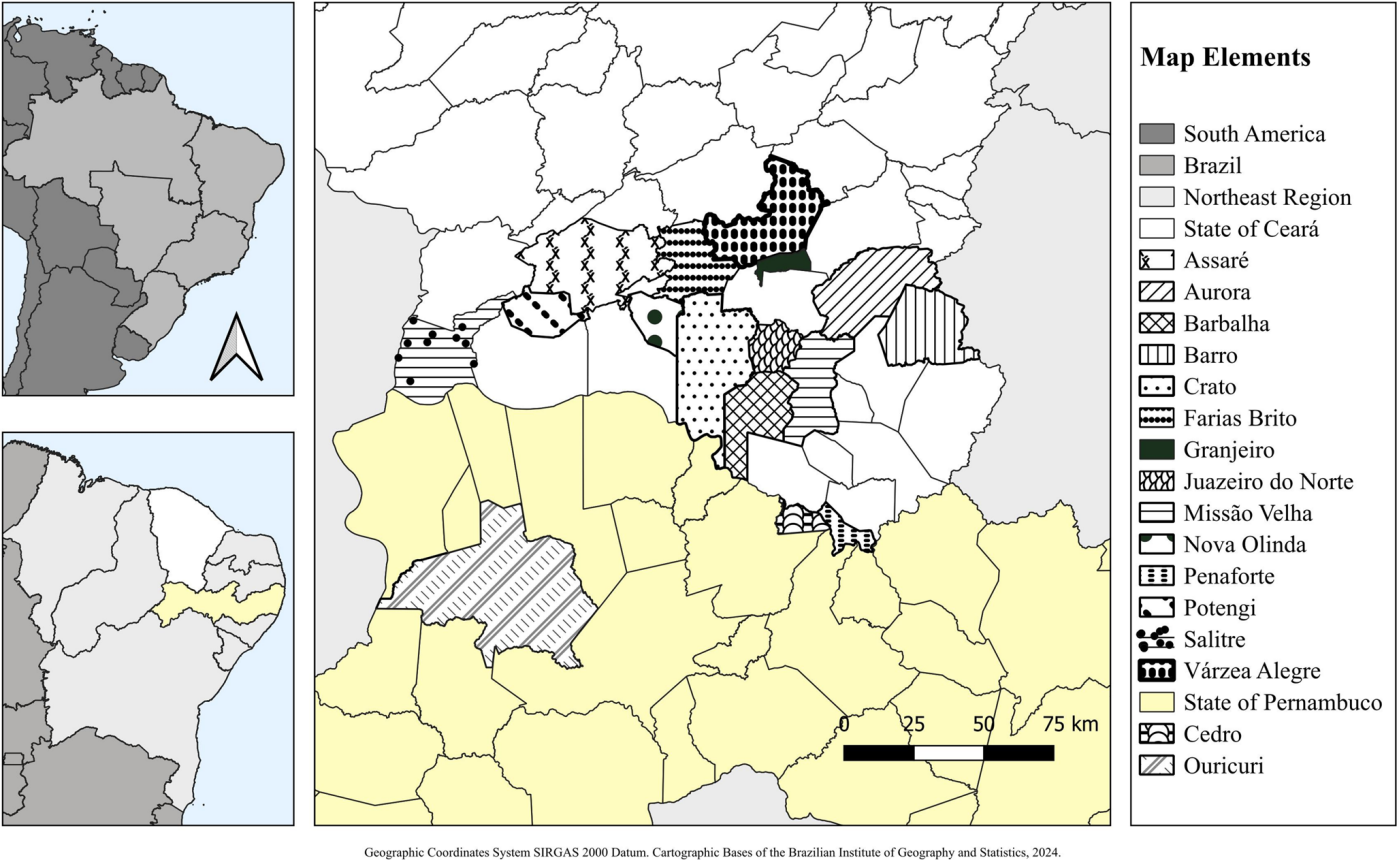
Geographic area where the research was conducted

The study evaluated bovine carcasses and viscera from January 2020 to December 2025 at a slaughterhouse located in Juazeiro do Norte, in the state of Ceará, Brazil (Fig. 1). Activities were conducted at the Frigorífico Industrial do Cariri, an establishment operating under the supervision of the State Inspection Service. All procedures involved the participation of Official Veterinary Inspectors. The geographical map presented in Fig. 1 was generated using QGIS software, based on publicly available geographic data.

### Sanitary inspection procedure

*Post-mortem* inspection was performed after complete evisceration. Viscera were condemned when organs presented alterations rendering them unfit for human consumption. Carcass condemnation occurred when lesions were identified as localized or systemic. *Post-mortem* inspection was carried out through visual examination, palpation, and strategic incisions in specific or altered sites. For the purposes of this study, lesions were classified as suggestive of bovine tuberculosis based on the presence of granulomatous inflammation, caseous necrosis, calcification, or a combination of macroscopic features indicating chronic and/or systemic infectious processes, particularly when involving typical predilection sites such as lungs and lymph nodes. In cases lacking classic caseous characteristics, inclusion was based on the overall distribution and pattern of lesions, consistent with generalized infectious disease as determined by official veterinary inspection criteria (Brasil 2017).

### Data curation

Official data were obtained from standardized inspection records and spreadsheets of the Agência de Defesa Agropecuária do Estado do Ceará (ADAGRI) at the slaughterhouse and organized into tables using Google Sheets software. These records follow official regulatory forms routinely used by veterinary inspection services, and no additional predesigned data collection form was developed specifically for this study. The analyzed variables included: age of animals; municipality and state of origin; average carcass weight; condemned viscera; type of condemnation (partial or total); and presence and description of macroscopic lesions suggestive of bovine tuberculosis.

### Statistical analysis

A retrospective observational design was adopted based on official slaughterhouse inspection records. Prevalence of bovine tuberculosis (BTB)-like lesions was calculated as the number of condemned carcasses divided by the total number of slaughtered cattle during the study period and expressed as percentage with 95% confidence intervals (95% CI).

Associations between BTB occurrence and sex were evaluated using the chi-square test (χ²). Odds ratios (OR) with 95% confidence intervals were calculated to estimate the strength of association. Temporal trends in annual prevalence were assessed using a binomial logistic regression model, with year included as a continuous predictor variable. Statistical significance was established at *p* < 0.05.

All analyses were performed considering the total population of slaughtered animals during the study period.

## Results

The overall prevalence of carcasses condemned due to lesions suggestive of bovine tuberculosis was low (Table 1), with no significant temporal variation throughout the study period. Most affected animals were older than 36 months.

**Table 1:** Absolute and relative frequency of total condemnations of bovine carcasses due to lesions suggestive of bovine tuberculosis identified at the slaughterhouse between 2020 and 2025

Year	Total Slaughtered (Absolute Frequency)	Total Condemnations (Relative Frequency / Percentage)
2020	12,655	5/0.04%
2021	10,326	4/0.04%
2022	10,033	4/0.04%
2023	8,356	5/0.06%
2024	16,074	5/0.03%
2025	21,874	13/0.06%
TOTAL	79,318	36/0.045%

It is noteworthy that, in the analysis of the origin of carcasses subjected to total condemnation (Table 2), a broad geographic distribution was observed, involving 16 municipalities (Fig. 1), predominantly located in the state of Ceará, as well as in bordering areas of the state of Pernambuco.

**Table 2:** Origin of slaughtered animals, distribution by sex, and carcass weight of animals condemned due to lesions suggestive of bovine tuberculosis from 2020 to 2025

Municipality – State	Total Condemned Carcasses	Total Condemned Males	Carcass Weight (kg)	Total Condemned Females	Carcass Weight (kg)
Assaré - CE	1	-	-	1	256
Aurora - CE	2	1	142	1	239
Barbalha - CE	3	-	-	3	170
					267
					240
Barro - CE	2	-	-	2	206
					138
Cedro - PE	1	1	315	-	-
Crato - CE	7	3	166	4	170
			197		279
			147		120
					103
Farias Brito - CE	2	-	-	2	172
					203
Granjeiro - CE	1	-	-	1	217
Juazeiro do Norte - CE	2	1	248	1	238
Missão Velha - CE	4	3	152	1	234
			300		
			240		
Nova Olinda - CE	3	1	187	2	355
					323
Ouricuri - PE	1	1	307	-	-
Penaforte - CE	1	1	154	-	-
Potengi - CE	1	1	128	-	-
Salitre - CE	1	-	-	1	145
Várzea Alegre - CE	4	1	170	3	184
					128
					268
Total	36	14	2,853	22	4,655

Among the municipalities analyzed, Crato–CE showed the highest frequency of cases, followed by Missão Velha–CE and Várzea Alegre–CE (Table 2). Regarding sex distribution, females showed a higher proportion of cases compared to males. A statistically significant association was observed between sex and the occurrence of BTB-like lesions (*p* < 0.001), with females presenting higher odds of condemnation than males.

Analysis of the dataset revealed a high degree of similarity among the condemned animals, particularly regarding macroscopic lesions. Most cases were characterized by generalized infectious processes, including purulent, caseous, and granulomatous lesions, often accompanied by adhesions between viscera. The lesions primarily affected the lungs, mediastinal lymph nodes, diaphragm, liver, heart, and thoracic and abdominal cavities, representing classical findings compatible with bovine tuberculosis, including cases of miliary tuberculosis (Table 3). In a small number of cases, lesions classified as septicemia or suppuration did not present the typical caseous appearance; however, they were included as suggestive of bovine tuberculosis due to their systemic distribution, association with multiple affected organs, and overall macroscopic pattern compatible with generalized infectious disease, as assessed by official inspection criteria.

**Table 3:** Distribution of macroscopic lesions identified in condemned carcasses and viscera, according to sanitary inspection criteria

Diagnostic Category	Observed Macroscopic Lesions	Number of Animals	Relative Frequency (%)
Chronic Generalized Tuberculosis	Presence of caseous lesions, necrosis, and calcification in multiple organs.	14	38.89%
Polyserositis (Adhesions)	Chronic inflammation with fibrous adhesions between viscera (lung–rib, liver–diaphragm).	13	36.11%
Miliary Tuberculosis	Acute dissemination with multiple small nodules (granules) in lungs and viscera.	4	11.11%
Septicemia / Suppuration	Purulent, foul-smelling, and septicemic infectious processes without specific caseous features.	3	8.33%
Tuberculosis (Unspecified)	Diagnosis confirmed without detailed lesion description	2	5.56%
TOTAL	—	36	100%

**Fig. 2:**
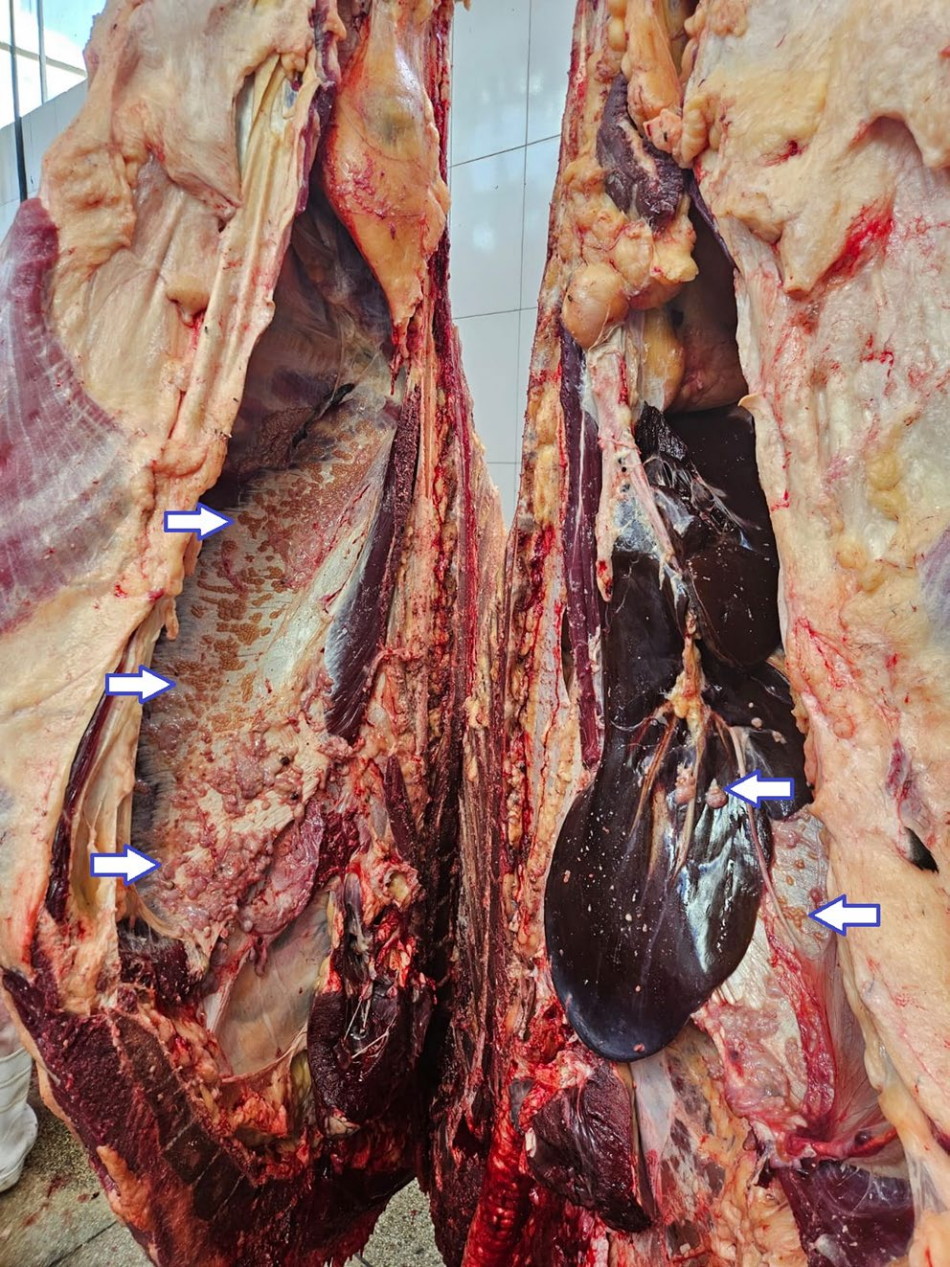
Bovine carcass and liver with macroscopic lesions compatible with bovine tuberculosis. Granulomatous pleuritis is evident, characterized by multiple firm caseous nodules adhered to the parietal pleura, suggestive of a chronic infectious process

**Fig. 3:**
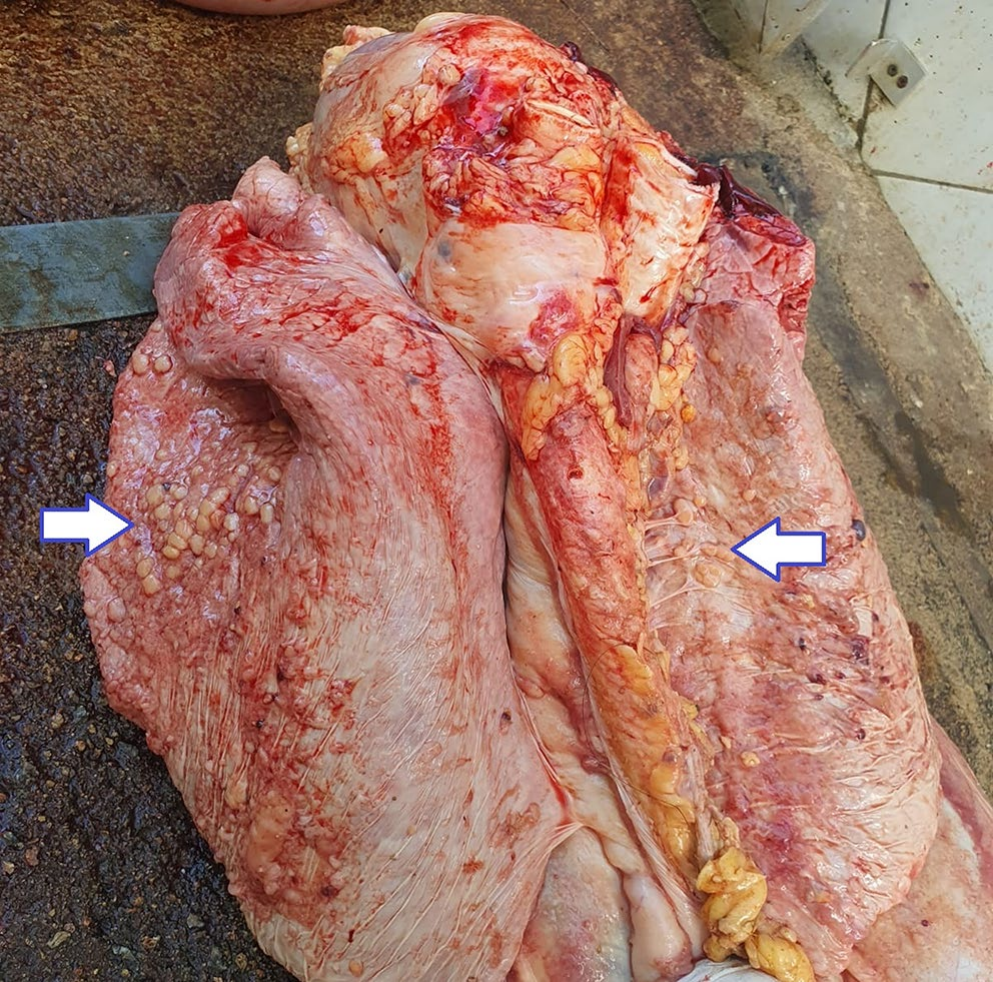
Bovine lungs with macroscopic lesions compatible with bovine tuberculosis. Granulomatous lesions typical of tuberculosis are evident on the lung serosa, characterized by multiple firm caseous nodules adhered to the visceral pleura, suggestive of a chronic infectious process

**Fig. 4:**
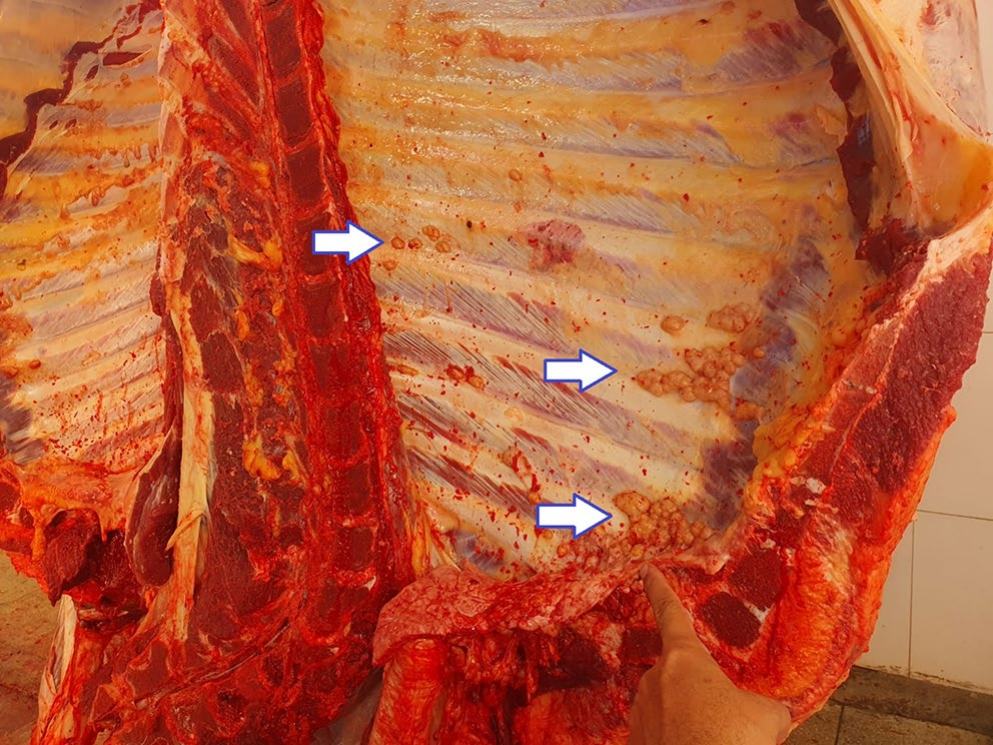
Bovine thoracic cavity showing firm nodules (granulomas) adhered to the parietal pleura, a lesion compatible with bovine tuberculosis

**Fig. 5:**
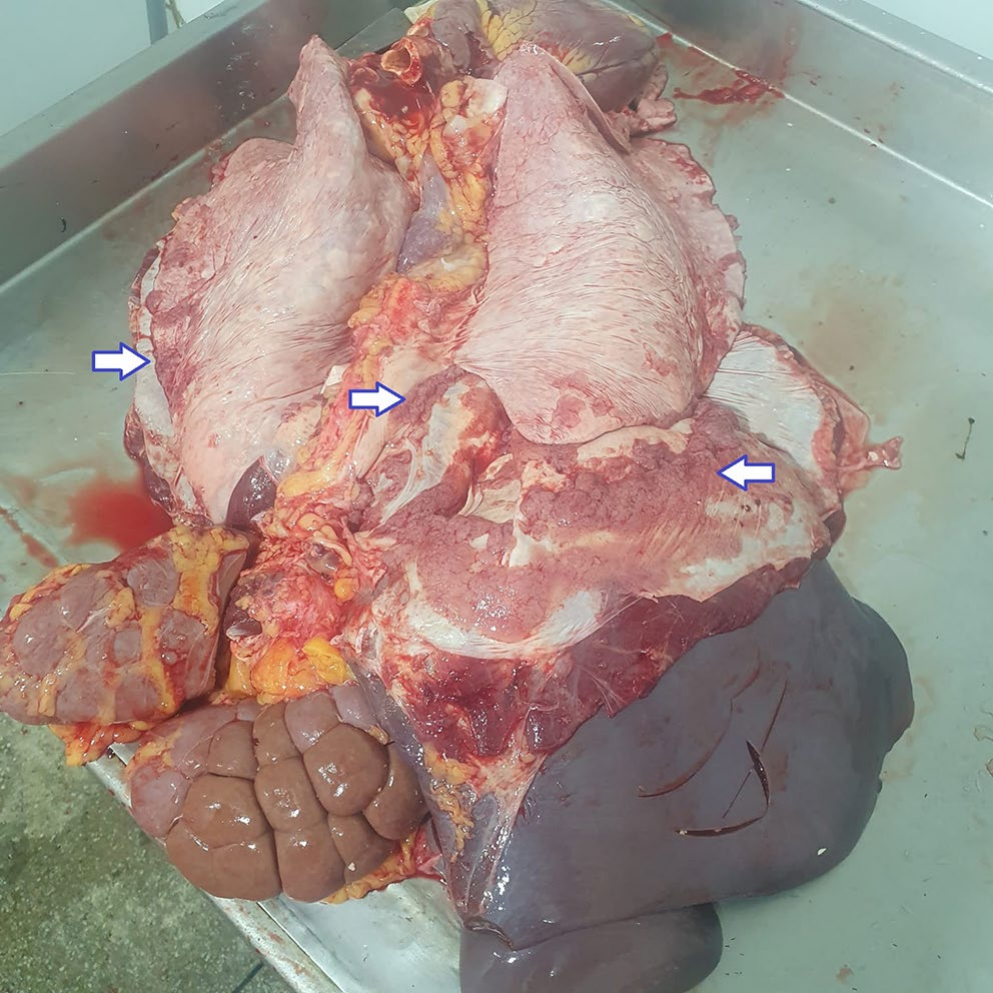
Total condemnation of viscera. Red viscera, including lungs, trachea, diaphragm, liver, and heart, show granulomatous lesions compatible with bovine tuberculosis. Alterations are more pronounced in the lungs and diaphragm muscle, indicating systemic dissemination and justifying full condemnation

Various macroscopic findings were observed in bovine carcasses and viscera affected by lesions compatible with bovine tuberculosis. Notable alterations were identified in the carcass, thoracic cavity, liver (Fig. 2), and lungs (Fig. 3), including the presence of granulomatous pleuritis and multiple firm caseous nodules attached to the parietal pleura (Fig. 4), as well as disseminated granulomas, indicating chronic progression and systemic spread of the infection (Fig. 5). The overall pattern of lesions observed, in accordance with Brazilian legislation, justifies the total condemnation of the carcass and viscera, following the criteria established by Brazilian sanitary inspection.

## Discussion

The frequency of 0.045% (36/79,318) of total condemnations due to lesions suggestive of bovine tuberculosis observed in this study indicates a low occurrence of the disease among the slaughtered animals during the analyzed period. However, despite this low percentage, the continuous detection of cases throughout all years evaluated (2020–2025) demonstrates the persistence of the agent within the source herds, evidencing that the disease remains active in the study area. This finding reinforces the endemic and silent nature of bovine tuberculosis, which is often identified only during *post-mortem* inspection (Ferreira et al. 2020).

The present study identified a low prevalence (0.045%) of carcasses condemned due to lesions suggestive of bovine tuberculosis (BTB) during a six-year period of official slaughterhouse inspection. Although numerically low, the consistent annual detection of cases indicates persistent endemic circulation of agents compatible with bovine tuberculosis in the region. Similar low but stable prevalence rates have been reported in slaughterhouse-based studies conducted in endemic tropical areas (Garcia et al. 2021; WOAH 2022).

Slaughterhouse surveillance predominantly detects animals with advanced or generalized lesions, as early or latent infections may not present visible macroscopic changes (Vicenzi et al. 2025). Therefore, the prevalence observed in this study (0.045%) likely underestimates the true infection burden at herd level, which may be considerably higher than that detected through routine meat inspection.

This limitation becomes apparent when comparing regional data from the Brazilian National Program for the Control and Eradication of Brucellosis and Tuberculosis (PNCEBT). Brazilian states such as Bahia (Northeast region) and Mato Grosso do Sul (Central-West region) are classified as Class A, with a prevalence of tuberculous outbreaks below 2%. In contrast, Pernambuco is classified as Class B, with an estimated prevalence of tuberculous outbreaks between 2% and < 3%, reflecting moderate surveillance coverage and more reliable detection. Meanwhile, states like Ceará fall into Class E, with unknown prevalence due to insufficient testing and limited epidemiological data. These differences suggest that the low prevalence observed in abattoir-based studies, particularly in regions with limited surveillance, may reflect gaps in detection rather than genuinely low infection rates (Brasil, 2025). Nevertheless, post-mortem inspection remains a critical complementary surveillance strategy, especially in areas where systematic tuberculin testing is limited (WOAH 2022; Vera-Salmoral et al. 2024).

The predominance of cases in animals older than 36 months is consistent with the chronic and progressive nature of BTB (Broughan et al. 2016). Prolonged exposure within herds increases the cumulative risk of infection and the development of lesions (Rinchen et al. 2026). Females accounted for a higher proportion of cases, and a statistically significant association between sex and BTB-like lesions was observed (χ² = 17.71; *p* < 0.001), with females showing markedly higher odds of condemnation compared to males (OR = 5.76; 95% CI: 2.92–11.37). Similar findings have been reported in previous studies, in which higher frequencies of bovine tuberculosis in females were attributed primarily to management and production-related factors rather than intrinsic biological susceptibility (Broughan et al. 2016; Neill et al. 2005). In many production systems, females (particularly cull cows from dairy or mixed systems) remain in herds for longer periods, increasing cumulative exposure to infectious agents. Additionally, management conditions typical of dairy production, such as higher animal density, prolonged confinement, and repeated handling, may facilitate transmission within these populations. Therefore, the observed association between sex and condemnation risk likely reflects differences in production systems and length of stay in the herd rather than a sex-specific predisposition to infection.

Lesions were predominantly granulomatous with caseous necrosis and calcification, mainly affecting lungs and mediastinal lymph nodes. This anatomical distribution reinforces inhalation as the primary transmission route (Neill et al. 2005). Hepatic involvement observed in some animals may indicate hematogenous dissemination in advanced cases. These macroscopic findings are consistent with classical pathological descriptions of bovine tuberculosis (WOAH 2022) and support the reliability of routine meat inspection for identifying characteristic chronic lesions.

From an economic perspective, even low prevalence levels can generate cumulative losses due to carcass condemnation and organ rejection, particularly in high-throughput slaughter systems. Additionally, the zoonotic potential of *M. bovis* reinforces the importance of strict inspection protocols and continuous surveillance to mitigate public health risks (Devi et al. 2021).

The absence of a significant temporal trend suggests epidemiological stability throughout the study period. However, stable occurrence does not equate to effective control, but rather to persistent endemic maintenance. Strengthening farm-level control measures, improving traceability systems, and integrating slaughterhouse data into regional surveillance networks are essential steps toward reducing infection sources and interrupting transmission cycles (Cespedes et al. 2021).

Within a One Health framework, the integration of veterinary inspection services with public health authorities enhances the epidemiological value of abattoir data. Slaughterhouse monitoring should therefore be considered a strategic component of bovine tuberculosis control programs in tropical production systems. Overall, despite the low detected prevalence, the consistent identification of characteristic lesions demonstrates ongoing pathogen circulation and highlights the importance of systematic post-mortem inspection as a cost-effective epidemiological surveillance tool.

This study presents inherent limitations associated with the use of secondary data from post-mortem inspection records. Diagnosis was based exclusively on macroscopic evaluation, without laboratory confirmation through histopathology, bacterial culture, or molecular methods. Therefore, it was not possible to confirm the etiological agent responsible for the observed lesions, and they cannot be definitively attributed to Mycobacterium bovis. Other pathogens capable of producing granulomatous lesions cannot be completely ruled out. Thus, the findings of this study should be interpreted as lesions suggestive of bovine tuberculosis rather than confirmed cases. Consequently, misclassification bias cannot be excluded, and the true prevalence of infection may be either underestimated or overestimated due to the limited sensitivity and specificity of routine meat inspection, particularly in early or paucibacillary infections (Filho et al. 2019; Kelly et al. 2021; Neto et al. 2024).

Additionally, herd-level data and on-farm testing results were not available, limiting inference regarding infection dynamics at the source level. Furthermore, the study was based on data from a single slaughterhouse, and although animals originated from multiple municipalities, the findings reflect a local or regional epidemiological context and should not be generalized to the entire Northeastern region of Brazil. Despite these limitations, slaughterhouse surveillance remains a valuable epidemiological tool for detecting advanced and systemic cases, contributing to regional disease monitoring (García-Díez et al. 2023).

The study demonstrated the continuous occurrence of lesions suggestive of bovine tuberculosis over the evaluated years, with a predominance in females, highlighting the persistence of the disease in the region. Although the observed prevalence was low, the detection of advanced, chronic, and in some cases systemic (miliary) lesions indicates that infected animals are not being identified and removed at early stages at the farm level. This finding suggests failures in the effectiveness of current control measures, particularly in early detection and surveillance within herds. Therefore, these results reinforce the urgent need to strengthen on-farm control strategies, including periodic testing and strict monitoring of animal movement. In addition, improving animal traceability systems is essential to ensure that, once a condemned carcass is identified at slaughter, sanitary authorities can effectively trace back and implement control measures at the farm of origin. Simultaneously, post-mortem inspection is confirmed as a strategic tool within a One Health framework, and the records generated serve as an essential instrument for epidemiological surveillance and for guiding more effective bovine tuberculosis control policies. Future studies should adopt prospective approaches incorporating laboratory confirmation through bacterial culture and molecular techniques, including strain typing, to better characterize the epidemiology and circulating strains of Mycobacterium bovis in the Cariri region.

## Data Availability

Not applicable.
